# Status and Development of Nuclear Medicine Over One Decade in Beijing

**DOI:** 10.1055/s-0044-1778712

**Published:** 2024-04-11

**Authors:** Fei Luo, Jianhua Geng, Shengzu Chen

**Affiliations:** 1 Department of Radiation Oncology, Beijing Shijitan Hospital, Capital Medical University, Beijing, People's Republic of China; 2 Department of Nuclear Medicine, National Cancer Center/National Clinical Research Center for Cancer/Cancer Hospital, Chinese Academy of Medical Sciences and Peking Union Medical College, Beijing, People's Republic of China

**Keywords:** nuclear medicine practice, positron emission tomography, single-photon emission computed tomography, quality control, nuclear medicine personnel, nuclear medicine imaging

## Abstract

**Objective** Our objective was to investigate the basic information of the personnel and facilities of nuclear medicine in Beijing.

**Methods** This survey was performed by the Beijing Quality Control Center in 2018. The investigation included personnel, equipment, and clinical applications, and data were then compared with previous surveys. The paper questionnaires were used for the survey, which required information about the personnel, devices, and clinical applications.

**Results** About 38 nuclear medicine departments in Beijing were involved in the survey. The number of nuclear medicine staff was 531 in 2018, showing an increase of 58.7% over the past decade. Positron emission tomography/computed tomography (PET/CT), single-photon emission computed tomography (SPECT), and single-photon emission computed tomography/computed tomography (SPECT/CT) represented the main nuclear medicine facilities, and the total number of surveyed departments was 18, 24, and 34, respectively. The quality control results showed significant improvement from the 2005 levels. The total number of scintigraphy procedures was estimated at 199,607 (153,185 SPECT and 46,422 PET/CT). The estimated annual number of scintigraphy images was 8.9 per 1,000 population for SPECT and 2.7 per 1,000 population for PET/CT during 2018. The most frequent radioiodine-targeted therapy was ^131^ I-targeted therapy for hyperthyroidism in 2018.

**Conclusion** Nuclear medicine has experienced rapid growth in the past 10 years in Beijing, either in personnel, equipment, and scintigraphy. Future efforts will focus on the use of new isotopes in the diagnosis, implementing quality strategy, and enhancing training.

## Introduction

Over 60 years ago, the first laboratory of isotope was established in Beijing. Nuclear medicine has evolved into a specialized branch of medicine over the past half-century, characterized by advanced diagnostic and treatment techniques. The rapid development of diagnostic methods and equipment in recent years is expected to continue, resulting in greater accuracy and safety. The introduction of innovative instrumentation and radiopharmaceuticals accelerated its progress. 1 Notably, the extensive utilization of positron emission tomography/computed tomography (PET/CT) has enhanced the association between structural and molecular observations. Then, PET/magnetic resonance imaging that integrates structural, functional, and molecular imaging technology in certain clinical applications presents a promising “one-stop shop” approach. 2

As the economic and political center of China, Beijing is a thriving hub of medical care and one of the country's largest cities. Almost all large-scale hospitals in Beijing have established nuclear medicine departments. In 2018, a survey was conducted with a focus on staff, equipment, and clinical applications in the field of nuclear medicine. Additionally, a retrospective analysis was performed to provide a comprehensive overview of the current state of nuclear medicine practice. The aim of this study was to assess the advancements made by nuclear medicine departments and to estimate the overall situation in Beijing.

## Materials and Methods

To obtain the necessary information, questionnaires were dispatched to hospitals via email. Departments that did not respond to our inquiry were contacted by telephone to obtain necessary data. Hospitals containing a nuclear medicine department were requested to provide the following details:

- Basic information of personnel working in nuclear medicine.
- Number, models, and the age of each single-photon emission computed tomography (SPECT), single-photon emission computed tomography/computed tomography (SPECT/CT), PET, and PET/CT machine in current use.
- Categories of quality control (QC) performed conventionally (daily, weekly, monthly, quarterly, and yearly) and their proportion.
- Annual number of radioimmunoassay, frequency of main clinical applications for SPECT (or SPECT/CT) and PET/CT(or PET), and radioisotope therapies.
- Radiological protection measures.

The questionnaire on equipment and procedures asked for details of the imaging equipment available in each department and for the numbers of each type of diagnostic investigation or therapeutic treatment performed in the period from November 1, 2016 to October 31, 2017. A comprehensive analysis of the outcomes was conducted by comparing the current survey to previous surveys. 3 4 The data underwent two manual matching processes by the working group and were subsequently incorporated into the final statistical summary following verification. We sought to provide beneficial information on the contemporary nuclear medicine status and to analyze the prevailing trends of nuclear medicine practice in Beijing, through the comparison of four independent investigations.

## Results

In 2018, 38 out of 48 departments returning completed questionnaires. Ten military hospitals were excluded from the analysis due to declined response. Previous surveys conducted in 2005, 2008, 2012, and 2015 included 30, 31, 27, and 33 departments, respectively.

### Personnel Status

The present study conducted an analysis of staffing patterns across various departments, revealing a total workforce of 531 individuals employed within the examined departments (as illustrated in Table 1 ). Staff distribution exhibited considerable variation across departments, with 14 departments (36.8%) employing fewer than 10 personnel, 15 departments maintaining a staff range of 10 to 20 individuals, and 4 major departments employing more than 30 staff members.

**Table 1:** General information of personnel from 1st to 5th survey in Beijing

Characteristic	2008	2012	2015	2018
Gender				
Male	139 (41.6%)	136 (40.8%)	181 (41.7%)	208 (39.2%)
Female	195 (58.4%)	197 (59.2%)	253 (58.3%)	323 (60.8%)
Total	334	333	434	531
Post				
Physician	143 (42.8%)	148 (44.4%)	191 (44.0%)	238 (44.8%)
Technician	133 (39.8%)	128 (38.4%)	159 (36.6%)	174 (32.8%)
Nurse	31 (9.3%)	26 (7.8%)	57 (13.1%)	80 (15.1%)
Physicist	4 (1.2%)	4 (1.2%)	6 (1.4%)	6 (1.1%)
Chemist	7 (2.1%)	10 (3.0%)	10 (2.3%)	23 (4.3%)
Engineer	4 (1.6%)	5 (1.5%)	1 (0.2%)	3 (0.6%)
Other ^a^	12 (3.6%)	12 (3.6%)	10 (2.3%)	7 (1.3%)
Education				
Doctor	37 (11.1%)	51 (15.3%)	97 (22.4%)	118 (22.2%)
Master	54 (16.2%)	75 (22.5%)	99 (22.8%)	104 (19.6%)
Bachelor	101 (30.2%)	111 (33.3%)	151 (34.8%)	205 (38.6%)
Under bachelor	142 (42.5%)	96 (28.8%)	87 (20.0%)	104 (19.6%)
Position				
Senior	78 (23.4%)	75 (22.5%)	99 (22.8%)	118 (22.2%)
Intermediate	137 (41.0%)	124 (37.2%)	156 (35.9%)	192 (36.2%)
Junior	119 (35.6%)	134 (40.2%)	179 (41.2%)	221 (41.6%)

Note: ^a^ Other includes persons employed through internships.

Moreover, the study identified that a mere six sites employed physicists, while nine sites had chemists on staff, and only a handful of departments had in-house engineers. In the field of nuclear medicine, there were 238 doctors employed, with 118 of them being specialists based in Beijing. Notably, the percentage of nurses increased from 6.9 to 15.1% during the study period. Prior to 2005, there were no chemists; however, by 2018, they constituted 4.3% of the workforce. Collectively, engineers, physicists, and chemists accounted for a mere 5% of the workforce.

In regard to educational qualifications, 22.2% of the respondents held a Doctor of Medicine and/or Doctor of Philosophy degree, while the majority of the staff possessed a bachelor's degree or equivalent, representing 58.2% of the sample. Notably, 19.6% of the staff had attained less than a bachelor's degree, typically comprising technicians and nurses who had completed vocational school or 3 years of education. The mean number of personnel per department was 13.9, indicating a 29.9% rise in contrast to the antecedent 2005 data. The majority of the workforce comprised medical physicians and technicians, representing as much as 77.6% in 2018, which remained unaltered over the previous decade.

### Equipment and Quality Control

**Fig. 1:**
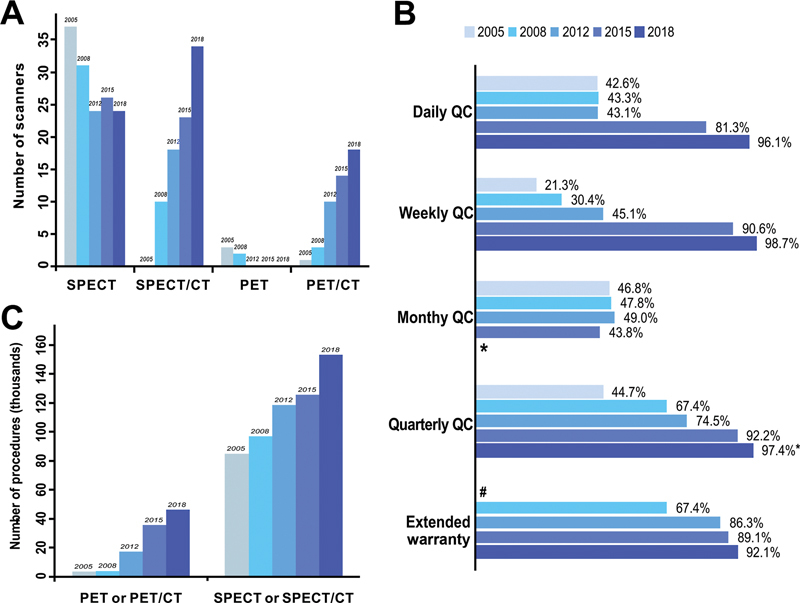
( **A** ) The changes in large-scale equipment based on survey year. ( **B** ) Chronological changes for the percentages of hospitals performing QC from 2005 to 2018. ( **C** ) The annual number of scans (thousands) using nuclear medicine in Beijing. QC, quality control; PET, positron emission tomography; PET/CT, positron emission tomography/computed tomography; SPECT, single-photon emission computed tomography; SPECT/CT, single-photon emission computed tomography/computed tomography. * In 2015, the Nuclear Medicine Quality Control Center in Beijing revised the equipment quality control standards, removing the requirement for monthly quality control. So, the standard of QC was daily, weekly, and semiannual in 2018. ^#^ Not involved in survey of 2005.

In the survey of 2018, 38 departments had installed 58 SPECT and /or SPECT/CT scanners (mean 1.5). No simple gamma cameras were in use. The number of hybrid SPECT/CT scanners accounted for 58.6%. PET/CT scanners increased from 1 to 18, and no simple PET scanners were in use, while five facilities had installed cyclotrons. Overall, 19 departments were equipped with only one scanner, which was usually a SPECT and /or SPECT/CT machine. A large hospital, which had the greatest number of scanners (for a total of eight), installed two PET/CT machines. As shown in Fig. 1A , there is a brief comparison of large-scale equipment during different survey periods.

More than 90% of scanners were submitted for routine QC analysis in 2018. In the previous four surveys, the QC process included daily, weekly, monthly, and quarterly. However, the standard was improved to daily, weekly, and semiannual QC in 2018. Few of the equipment did not undergo any QC work, which was different from 2005, when most hospitals did not conventionally perform QC procedures. The proportion of QC and extended warranty service was increased by year ( Fig. 1B ).

### Clinical Applications

The total number of nuclear medicine scintigraphy images for the 38 hospitals was 199,607, including 153,185 (76.7%) for 58 SPECT, and 46,422 (23.2%) for 18 PET/CT in 2018. The average number of images for each device was 2,641 for SPECT. However, the annual imaging figure was fewer than 1,000 at five sites. The average number of images for each PET/CT was 2,579. The annual number of scans included in previous surveys is shown in Fig. 1C . The frequently performed applications are listed in Table 2 .

**Table 2:** The most frequently performed applications from survey in Beijing

Examined sites	Radionuclide	Radionuclide-conjugated drug
Bone	^99^ Tc ^m^	MDP
Brain	^99^ Tc ^m^	ECD
	^18^ F	FDG
	^11^ C	NMSP
Cancer	^18^ F	FDG
Heart	^99^ Tc ^m^	RBC
	^99^ Tc ^m^	MIBI
	^99^ Tc ^m^	FDG
Lung	^99^ Tc ^m^	MAA
	^99^ Tc ^m^	DTPA
Liver	^99^ Tc ^m^	RBC
Lymph node	^99^ Tc ^m^	Dx
Renal	^99^ Tc ^m^	DTPA
Thyroid	^99^ Tc ^m^	
Parathyroid	^99^ Tc ^m^	MIBI

Abbreviations: DTPA, diethylenetriaminepentaacetic acid; Dx, dextran; ECD, ethyl cysteinate dimer; FDG, fluorodeoxyglucose; MAA, macroaggregated albumin; MDP, methylenediphosphonate; MIBI, methoxyisobutylisonitrile; NMSP, N-methylspiperone; RBC, red blood cell.

In the 2018 survey, only 11 nuclear medicine departments maintained in vitro studies, whereas 16 departments had performed these studies in the previous four surveys. A total of 2.0 million in vitro studies were performed in 2018. Notably, one site had the highest number of examination items, totaling 46, while the lowest number of items was a mere 2. A collective of 31 institutions administered a total of 6,509 therapeutic interventions ( Table 3 ).

**Table 3:** Number and details of radionuclide therapy in 2018

	Cases	Rate (%)	Number of departments
^131^ **I for hyperthyroidism**	2,971	46.3	21
^131^ I for thyroid cancer	1,632	25.4	6
^99^ Tc-MDP for arthritic	921	14.3	6
^125^ I seeds implantation	469	5.0	5
^153^ Sm-EDTMP and ^89^ SrCl _2_ for bone metastasis	328	3.6	19
^131^ I-MIBG therapy for neuroblastoma	100	1.5	1

## Discussion

According to the results of this survey and previous surveys, we can see that nuclear medicine has gradually developed in Beijing. In terms of the per capita availability of nuclear medicine specialists, the data indicate an average of one specialist per 145,000 individuals, a figure slightly higher than that observed in Poland but lower than the European average. 5 6 From the perspective of personnel composition, the increase in the proportion of nurses may be attributed to a more detailed division of labor, and this increase in nurses likely contributes to an improved quality of medical care. The proportion of chemists have also been steadily rising, primarily driven by the growing number of installed PET/CT machines. Despite the rising proportions of chemists, physicists, and engineers, it becomes evident that this expansion may still not suffice to meet the demands of the field. Therefore, hospitals and universities should consider creating more positions to actively attract and engage candidates with backgrounds in pharmacy/chemistry and physics/engineering. 7

In terms of gender, women's representation increased over time. 8 9 10 In terms of education level, the staffs of nuclear medicine have made significant advances. All of the hospitals in Beijing were implemented residency training programs, similar to other countries. 11 12 Nuclear medicine in China has established training rules and regulations that draw on international experience, particularly those related to combining training in both radiology and nuclear medicine. 13 14 In some minority subspecialty (e.g., for pediatric nuclear medicine), nuclear medicine physicians have to be trained and regularly receive continuing medical education in this field. 12 15 16 17 18

In 2005, the utilization of hybrid SPECT/CT was nonexistent in 2005. However, a decade later, 50% of the surveyed sites reported operating one to two SPECT/CT systems, which is lower than the figures reported in the Wieder study. Notably, the majority of centers do not fully utilize the diagnostic potential of CT components. In contrast, the number of PET/CT instruments grew rapidly, this growth was 250.0% in the first three surveys before 2015, and the latest survey showed a further 28.5% increase. A total of 15 institutes had installed hybrid PET/CT scanners, and only five facilities had installed cyclotrons, which resulted in significantly lower frequencies in comparison to other countries. 11

Thanks to the staff training and the organization of supervision, the status of quality assurance and QC was improved during the past years. The purpose of QC is to ensure that equipment or processes adhere to standardized practices. The essence of quality assurance is assurance, a degree of assurance or confidence. The QC of equipment have now become a routine practice to ensure the consistency of use in the nuclear medicine of Beijing. To ensure wider dissemination and use of published procedure standards, appropriate use criteria must be developed and implemented. 19 By focusing on quality, patients receive diagnosis and treatment that is more safe, accurate, reliable, available, and integrated.

Both diagnostic and therapeutic procedures are important in nuclear medicine. In Beijing, scintigraphy appears to be the most important approach and contributes to the most growth. Because 10 military hospitals in Beijing that did not respond to our survey, we estimated that 40,312 SPECT procedures had been performed in them. The total number of SPECT procedures was 193,497, equivalent to 8.9 per 1,000 people, an increase of 32.8% from 2005. 3 4 For PET studies, the number of departments and studies clearly increased during the study period. The total number of procedures for PET/CT was estimated 66,834 for 28 PET/CT devices, with approximately 3.0 per 1,000 people during 2018. Considering the floating populations in large cities seeking medical attention, we may have overestimated the actual number.

There have been few radionuclide-targeted therapy advances over the past years. By contrast, institutes that owned facilities for radionuclide-targeted therapy increased by 36.8%, and the annual treatment rate increased 2.6-fold in 10 years. There should be more exploration of possible resources in nuclear medicine for use as radiopharmaceuticals in the field of theranostics or therapy. Nuclear medicine imaging is an essential component of the in vivo companion diagnostics for cancer therapy, and other noninfectious diseases that are constantly changing and being developed. It is the way that nuclear medicine is moving forward in the future, regardless of developed or developing countries. 7

The survey has some limitations. Although we have list the most frequently performed applications, the survey lacks data regarding the specific frequencies of each examination. This limitation may impact our understanding of the actual frequency of patients undergoing nuclear medicine examinations. It may also prevent us from providing in-depth insights into the indications for nuclear medicine examinations and clinical decision-making. Therefore, when interpreting our research results, it is crucial to consider these limitations cautiously. It's also important to emphasize that future research can address these shortcomings by collecting more detailed data to gain a more comprehensive insight, thereby better guiding the application and decision-making in nuclear medicine.

## Conclusion

There are many factors that influence nuclear medicine's prosperity in various countries. The success of nuclear medicine over the past decades in Beijing was based on the new instruments developed and installed widely in many institutions, and of course the good availability of radiopharmaceuticals. Although strides have been made, some important issues need to be further addressed. In the future, we will be focusing on the use of new isotopes in the diagnosis and treatment of cancer and other noninfectious diseases, implementing the quality strategy and strengthening the training program for nuclear medicine personnel. Meanwhile, we should strive to promote global cooperation.
